# AIM2/IL-1α/TGF-β Axis in PBMCs From Exacerbated Chronic Obstructive Pulmonary Disease (COPD) Patients Is Not Related to COX-2-Dependent Inflammatory Pathway

**DOI:** 10.3389/fphys.2019.01235

**Published:** 2019-10-01

**Authors:** Antonio Molino, Michela Terlizzi, Chiara Colarusso, Antonietta Rossi, Pasquale Somma, Alessandro Saglia, Aldo Pinto, Rosalinda Sorrentino

**Affiliations:** ^1^Department of Respiratory Medicine, Respiratory Division, University of Naples Federico II, Naples, Italy; ^2^Department of Pharmacy, University of Salerno, Fisciano, Italy; ^3^Ph.D. Program in Drug Discovery and Development, Department of Pharmacy, University of Salerno, Fisciano, Italy; ^4^Department of Pharmacy, School of Medicine and Surgery, University of Naples Federico II, Naples, Italy; ^5^Department of Anatomy and Pathology, Ospedale dei Colli “Monaldi-CTO”, Naples, Italy

**Keywords:** lung, chronic lung inflammation, COPD, inflammasome, IL-1-like cytokines

## Abstract

Chronic obstructive pulmonary disease (COPD) is a lung disorder characterized by persistent respiratory symptoms and progressive airflow limitation as a consequence of a chronic inflammatory response. Corticosteroids are the main treatment for COPD patients with a history of exacerbation, in that they attenuate exacerbation and dyspnea, and improve the response to bronchodilators. Nevertheless, despite corticosteroid administration, COPD patients still undergo exacerbation phases. In this context, the aim of this study was to evaluate the activity of Absent in melanoma 2 (AIM2) inflammasome-dependent pathways under corticosteroid treatment during COPD exacerbation. Stable and exacerbated COPD-derived Peripheral Blood Mononuclear Cells (PBMCs) were treated with a well-known anti-inflammatory agent, Dexamethasone (DEX), in the presence or not of Poly (deoxyadenylic-deoxythymidylate) acid (Poly dA:dT), an AIM2 ligand. We found that IL-1α was highly increased when AIM2 was activated from Poly dA:dT in exacerbated, but not in stable, COPD-derived PBMCs. To note, the release of IL-1α after the stimulation of AIM2 in PBMCs obtained from stable (hospitalized) COPD patients was not higher from the basal conditions, though it was still as high as that observed for Poly dA:dT-stimulated PBMCs obtained from exacerbated patients. This effect was associated with a higher expression of AIM2 in pair-matched circulating CD14^+^ cells obtained from hospitalized patients who passed from the exacerbation to stable status. Because the difference between stable and exacerbated COPD patients relies on the treatment with corticosteroids, exacerbated and stable COPD-derived PBMCs were treated with DEX. Indeed, the release of IL-1α and TGF-β was not altered after DEX treatment. In conclusion, we found that the administration of DEX *in vitro* on exacerbated COPD-derived PBMCs was not able to revert the detrimental inflammatory mechanism associated with AIM2 activation responsible for the release of IL-1α and the ensuing TGF-β, contributing to the severity of disease.

## Introduction

Chronic Obstructive Pulmonary Disease (COPD) is a chronic inflammatory lung disease affecting approximately 10% of individuals older than 45 years worldwide (Barnes, 2016). The first risk factor of COPD is tobacco smoking, though only 15–20% of chronic smokers may develop clinically apparent pathology (Colarusso et al., 2017).

The clinical picture of COPD patients is characterized by chronic bronchitis, emphysema, shortness of breath and increased cough, due to mucus hypersecretion, airway obstruction and tissue remodeling (De Nardo et al., 2014), all symptoms associated with pulmonary chronic inflammation that leads to a progressive and irreversible decline of lung function (Sugimoto et al., 2016). The infiltration of immune cells such as neutrophils, macrophages, CD4^+^ and CD8^+^ lymphocytes, B cells, and other inflammatory cells into the small airways contributes to the pathogenesis of COPD through the release of several factors (e.g., cytokines, proteases and growth factors), resulting in tissue injury and airspace enlargement, hallmarks of this pathology (Borchers et al., 2007; Huang et al., 2017; Wang Y. et al., 2018). Current therapeutic options for COPD patients tend to cure the symptoms of the pathology. Corticosteroids, well-known immune-modulators, are routinely used (Miravitlles et al., 2016), decreasing exacerbation and dyspnea, and improving the response to bronchodilators (Tashkin and Strange, 2018). Indeed, the combination of corticosteroids and long-acting β_2_-agonists (LABAs) bronchodilators provides less exacerbations, ameliorating lung function and health status. However, the majority of patients are resistant to even high doses of inhaled or oral steroids (Barnes, 2013).

Several molecular mechanisms have been identified that might account for reduced steroid responsiveness and thus drug resistance, e.g., (i) reduced nuclear translocation of glucocorticoid receptor (GR); (ii) increased expression of GRβ; (iii) competition with the transcription factor activator protein 1; (iv) reduced expression of histone deacetylase 2 (Barnes, 2013). Moreover, corticosteroids remain non-effective in suppressing inflammation in that structural and inflammatory cells in the lung of COPD patients respond to cigarette smoke exposure and to other stimuli (e.g., environmental pollutants, pathogens), by releasing pro-inflammatory mediators and recruiting immune cells that contribute to the establishment of a chronic inflammatory microenvironment (Bhat et al., 2015; De Falco et al., 2017a), which is harmful for these patients.

Recent studies suggest that the multi-protein intracellular complex of inflammasome, a specialized signaling platform that governs pro-inflammatory cytokine release, may play a crucial role in contributing to the development of airway inflammation in COPD patients (De Falco et al., 2017a; Wang H. et al., 2018; Colarusso et al., 2019).

The inflammasome is a multiprotein complex that comprises the assembly of the nucleotide-binding domain, leucine-rich repeat (NLRs) or hematopoietic IFN-inducible nuclear antigens with 200 aa repeat (HIN200) family receptors, capable of binding the adaptor apoptosis-associated speck-like protein (ASC), and capable of inducing the auto-cleavage of procaspase-1, that in its active form facilitates the activation and then the release of pro-inflammatory IL-1-like cytokines (Terlizzi et al., 2014). So far, all the studies on the involvement of inflammatory patterns in COPD focused on the Nod-like Receptor 3 (NLRP3) inflammasome, whose persistent activation and over-expression may promote chronic inflammation.

For the first time, to our knowledge, we recently found that Absent in melanoma 2 (AIM2) inflammasome, but not NLRP3 inflammasome, was involved in COPD exacerbation (Colarusso et al., 2019).

AIM2 is a cytosolic dsDNA-sensing inflammasome, a key player in host defenses and a guardian of cellular integrity, which deregulation can contribute to immune-linked diseases, such as auto-inflammatory and auto-immune disorders (Guo et al., 2018). Therefore, because we previously showed the involvement of AIM2 in COPD exacerbation, the main goal of this study was to understand the role of AIM2 under corticosteroids treatment from the exacerbation to the induced stable condition during the hospitalization of patients.

We found that the administration of Dexamethasone *in vitro* on exacerbated COPD-derived PBMCs was not able to revert the detrimental inflammatory mechanism associated with AIM2 activation responsible for the release of IL-1α and the ensuing TGF-β, contributing to the severity of the disease.

## Materials and Methods

### Human Samples

Blood was collected from the same patient at two different times: in an exacerbated COPD phase and in a stable (after hospitalization) COPD phase. Patients were recruited at the “Monaldi-Azienda Ospedaliera (AORN)-Ospedale dei Colli” Hospital in Naples, Italy, in accordance with the Review Board that approved the project and the patients’ informed consent. The experimental protocol was performed in accordance with the guidelines and regulations provided by the Ethical Committee of the “Monaldi-Azienda Ospedaliera (AORN)-Ospedale dei Colli” (protocol n. 604/2017). COPD patients were smokers or former smokers (Table 1). Pharmacological treatment and the characteristics of COPD patients are available in Table 1. The mean age of enrolled patients was 50 ± 10 years old. Blood was collected and used within 24 h.

**TABLE 1:** COPD patients’ details.

**Patient**	**Gender**	**Age (years)**	**Smoking status**	**GOLD stage**	**Treatment**
#1	M	84	Former	3	Corticosteroid + LABA + LAMA
#2	M	72	Former	3	LABA + LAMA
#3	F	71	Former	2	LABA + LAMA
#4	M	63	Smoker	3	Corticosteroid + LABA + LAMA
#5	F	44	Smoker	1	Corticosteroid + LABA
#6	M	76	No Smoker	2	Corticosteroid + LABA + LAMA
#7	F	75	Former	2	Corticosteroid + LABA + LAMA
#8	M	69	Former	3	Corticosteroid + LABA + LAMA

*LABA, long-acting β2-agonist; LAMA, long-acting anti-muscarinic agent.*

### Isolation of Human PBMCs

Peripheral Blood Mononuclear Cells (PBMCs) were isolated according to Ficoll’s protocol as already reported (De Falco et al., 2017b). Specifically, blood (5 ml) was mixed with cell medium (RPMI, 5 ml), supplemented with sole antibiotics (Penicillin-Streptomycin, 1%), and Ficoll medium (Life Sciences, Italy). PBMCs layer was collected after centrifugation at 1125 *g* for 20 min. The remaining Ficoll solution was removed after centrifugation at 753 *g*. Platelets were separated from the PBMCs through centrifugation at 149 *g* for 10 min. PBMCs were then collected in a RPMI cell medium (supplemented with 1% Penicillin-Streptomycin and 10% Fetal Bovin Serum), plated and treated for 5 or 24 h. PBMCs were incubated with Poly dA:dT 1 μg/ml, Dexamethasone (DEX) 0.1 ng/ml and/or 1 ng/ml, Indomethacin (Indo) 3.5 μg/ml.

### Cytokine Measurements

IL-1α and TGF-β were measured in cell-free supernatants obtained from the PBMCs culture, respectively, after 5 and 24 h of treatment, using commercially available enzyme-linked immunosorbent assay kits (ELISAs) (eBioscience, CA, United States; R&D Systems, United States). Cytokine levels were expressed as pg/ml.

### PGE_2_ Quantification

Prostaglandin E_2_ was quantified according to the manufacturer’s instructions (Cayman chemicals BertinPharma, Montigny Le Bretonneux, France). PGE_2_ levels were expressed as pg/ml.

### Flow Cytometry Analysis

AIM2 expression was performed using flow cytometry (BD FacsCalibur Milan, Italy) by staining untreated PBMCs with the following antibodies: AIM2-FITC and CD14-PE (eBioscience, CA, United States).

## Statistical Analysis

Data are reported as the median±interquartile range. Statistical differences were assessed with a ONE-Way ANOVA followed by multiple comparison Dunn’s post–tests or Student’s *t*-tests (where appropriate) followed by a Mann–Whitney tests, as appropriate. *P*-values less than 0.05 were considered significant.

## Results

### Stable COPD-Derived PBMCs Released High Levels of IL-1α

**FIGURE 1:**
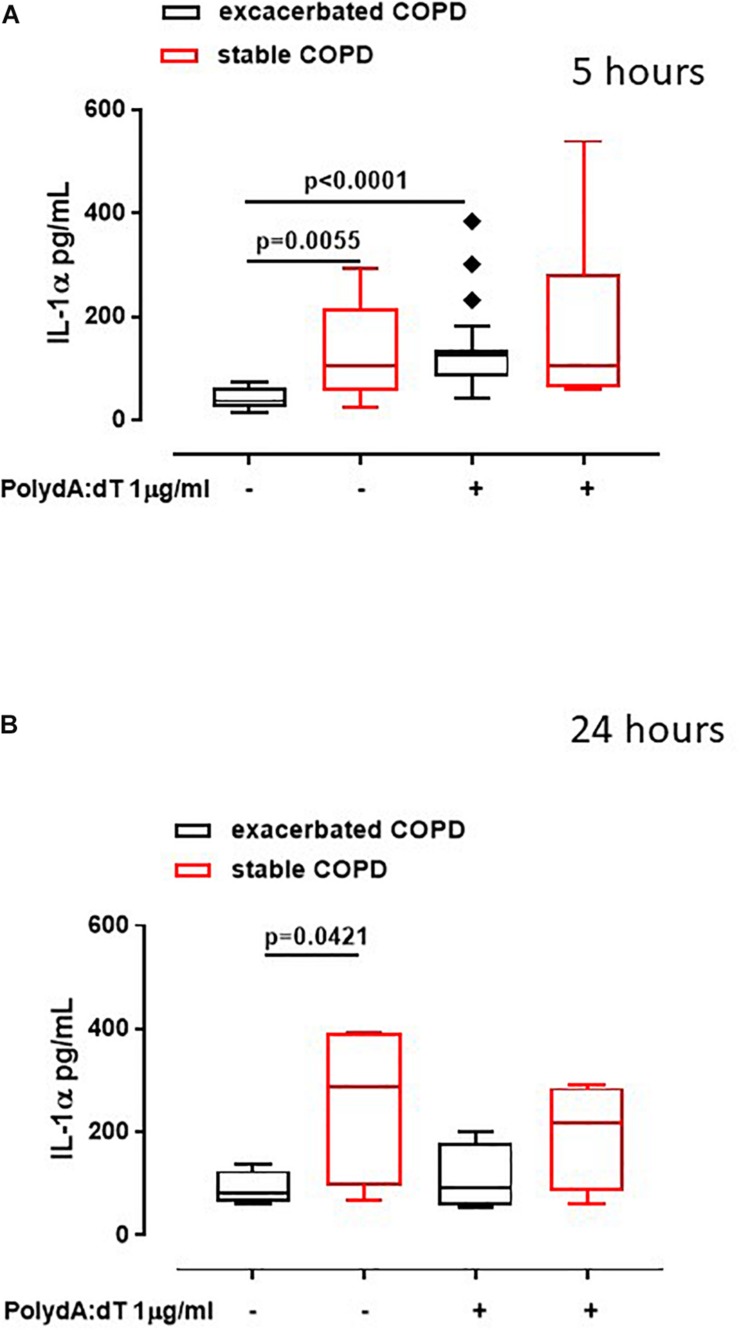
Exacerbated as well as stable COPD-derived PBMCs released high levels of IL-1α. Exacerbated and stable COPD patients-derived PBMCs were stimulated with an AIM2 ligand, Poly dA:dT (1 μg/ml) for 5 **(A)** and 24 h **(B)**. The activation of AIM2 significantly increased the release of IL-1α after 5 h of treatment from exacerbation (black bars) **(A)**, compared to stable COPD-derived PBMCs (red bars) **(A)**. After 24 h of treatment, IL-1α levels were higher in stable (red bars) **(B)**, compared to exacerbated patients (black bars) **(B)**, both at baseline and after AIM2 triggering, although no statistical differences were noted. Data are represented as median ± interquartile range (*n* = 17). Statistically significant differences were determined by ONE-way ANOVA followed by Dunn’s post–test.

**FIGURE 2:**
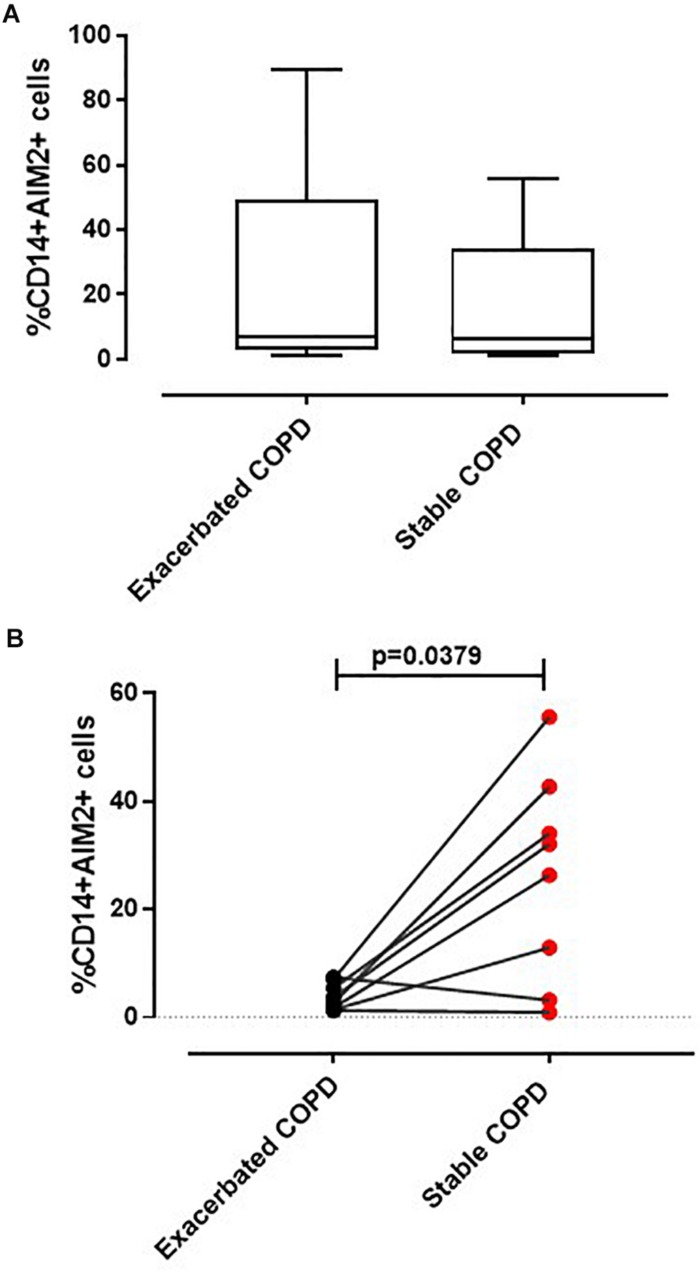
Expression of AIM2 in CD14^+^ PBMCs. Flow cytometry analysis was performed on CD14^+^ cells to evaluate AIM2 expression. **(A)** No differences in AIM2 expression were detected between CD14^+^ PBMCs derived from exacerbated and stable COPD patients. **(B)** Pair-matched samples (*n* = 8) showed an increased AIM2 expression in stable compared to the corresponding exacerbated condition COPD-derived PBMCs. Data are represented as median ± interquartile range. Statistically significant differences were determined by Mann–Whitney U test.

Recently we reported that the AIM2 inflammasome pathway was activated in exacerbated COPD-derived PBMCs and was responsible for IL-1α and TGF-β release in a caspase-1- and caspase-4-dependent manner (Colarusso et al., 2019). Although the involvement of inflammation during the stable phase of COPD is known (Fujimoto et al., 2005), the role of the inflammasome still requires clarification. To understand the involvement of the AIM2 inflammasome during different phases of COPD, PBMCs isolated from COPD patients during an exacerbated and subsequently during a stable phase (after hospitalization) were stimulated with an AIM2 inflammasome ligand, Poly dA:dT (1 μg/ml) for 5 (Figure 1A) and 24 h (Figure 1B). The activation of AIM2 by Poly dA:dT significantly increased the release of IL-1α in exacerbated COPD-derived PBMCs (Figure 1A, black bars). In contrast, we did not observe any statistical difference in terms of IL-1α release after Poly dA:dT stimulation of PBMCs isolated from patients during the stable phase of the disease (Figure 1A, red bars). Surprisingly, during basal conditions the release of IL-1α from PBMCs obtained from stable COPD patients (median ± SEM: 127.9 ± 28.2) was significantly higher than that released from Poly dA:dT-stimulated exacerbated COPD-derived PBMCs (median ± SEM: 43.27 ± 4.43) (Figure 1A, red bars vs. black bars). In sharp contrast, the stimulation of AIM2 with Poly dA:dT did not induce a significant increase of IL-1α release from stable COPD-derived PBMCs (median ± SEM: 182.9 ± 55.1), but it was still as high as that released from PBMCs during the exacerbation phase after AIM2 stimulation (median ± SEM: 133 ± 15) (Figure 1A, red bars vs. black bars). The same effect was observed 24 h after AIM2 stimulation (Figure 1B). In addition, we found that the expression of AIM2 in CD14^+^ cells was not statistically different between exacerbated and stable COPD-derived PBMCs (Figure 2A). In this graph, we had more exacerbated samples than stable samples; but, when we analyzed paired samples we observed that the expression of AIM2 was higher in the stable than exacerbated COPD-derived PBMCs. In particular, we found that six out of eight stable COPD patients had higher levels of AIM2 in the CD14^+^ cells (Figure 2B), implying that corticosteroids treatment during hospitalization could increase its transcriptional levels. Interestingly, the increased AIM2 expression in stable COPD-derived PBMCs was not associated with a higher activity of AIM2 after stimulation with Poly dA:dT in terms of IL-1α release (Figures 1A,B, red bars), as instead observed for exacerbated patients (Figures 1A,B, black bars). Nevertheless, the levels of IL-1α were still as high as those released from PBMCs during the exacerbation phase (Figure 1).

### Corticosteroids Did Not Alter the Release of IL-1α After AIM2 Inflammasome Activation in Both Exacerbated and Stable COPD-Derived PBMCs

In order to understand the role of the AIM2/IL-1α axis in both stable and exacerbated COPD in our experimental conditions, and because the difference between stable and exacerbated COPD patients relies on corticosteroid treatment, that needs to be intravenously administered to hospitalized exacerbated patients, we moved on by analyzing the effect of corticosteroids on AIM2-dependent pathway. Therefore, we treated PBMCs with Poly dA:dT in the presence or not of Dexamethasone (DEX, 0.1 ng/ml and 1 ng/ml).

**FIGURE 3:**
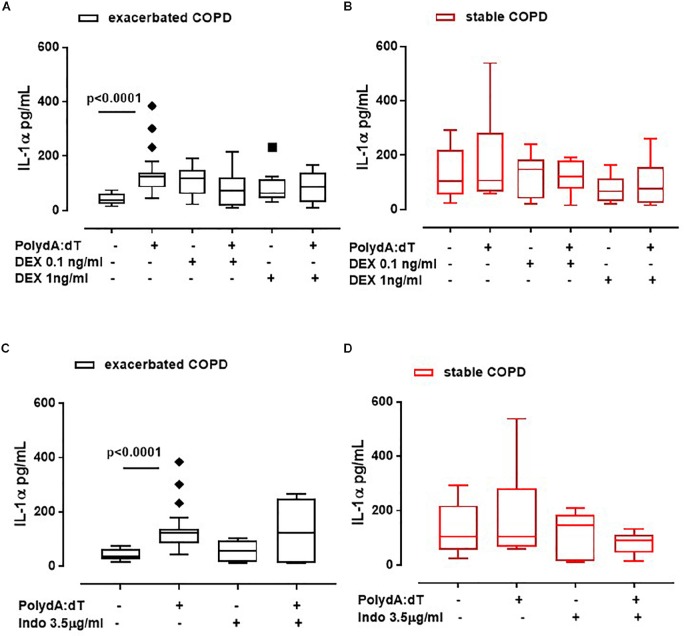
IL-1α release was not reverted after the treatment with anti-inflammatory agents. Exacerbated and stable COPD-derived PBMCs were stimulated with Dexamethasone (DEX, 0.1 ng/ml and 1 ng/ml) **(A,B)** and Indomethacin (Indo, 3.5 μg/ml) **(C,D)**, and IL-1α release was evaluated after 5 h of treatment. The addition of DEX did not alter IL-1α release from both exacerbated **(A)** and stable **(B)** COPD-derived PBMCs after AIM2 inflammasome triggering. IL-1α release was not reduced after Indomethacin (Indo, 3.5 μg/ml) addition to exacerbated **(C)** and stable **(D)** COPD-derived PBMCs in the presence or absence of Poly dA:dT (1 μg/ml). Data are represented as median ± interquartile range (*n* = 10). Statistically significant differences were determined by ONE-way ANOVA followed by Dunn’s post–test.

The administration of DEX did not alter the release of IL-1α from exacerbated COPD-derived PBMCs (Figure 3A). Surprisingly, these data were also observed for stable COPD-derived PBMCs (Figure 3B), implying that IL-1α release was not altered by the corticosteroid’s mechanism of action.

To better rule out COX-2 involvement, PBMCs were treated with a non-steroidal anti-inflammatory drug (NSAID), Indomethacin (Indo, 3.5 μg/ml). Again, IL-1α release was not altered when Poly dA:dT was co-administered with Indo to both exacerbated (Figure 3C) and stable (Figure 3D) COPD-derived PBMCs, implying that the treatment with classical anti-inflammatory agents does not modify the AIM2 inflammasome-dependent inflammatory pathway in COPD patients.

**FIGURE 4:**
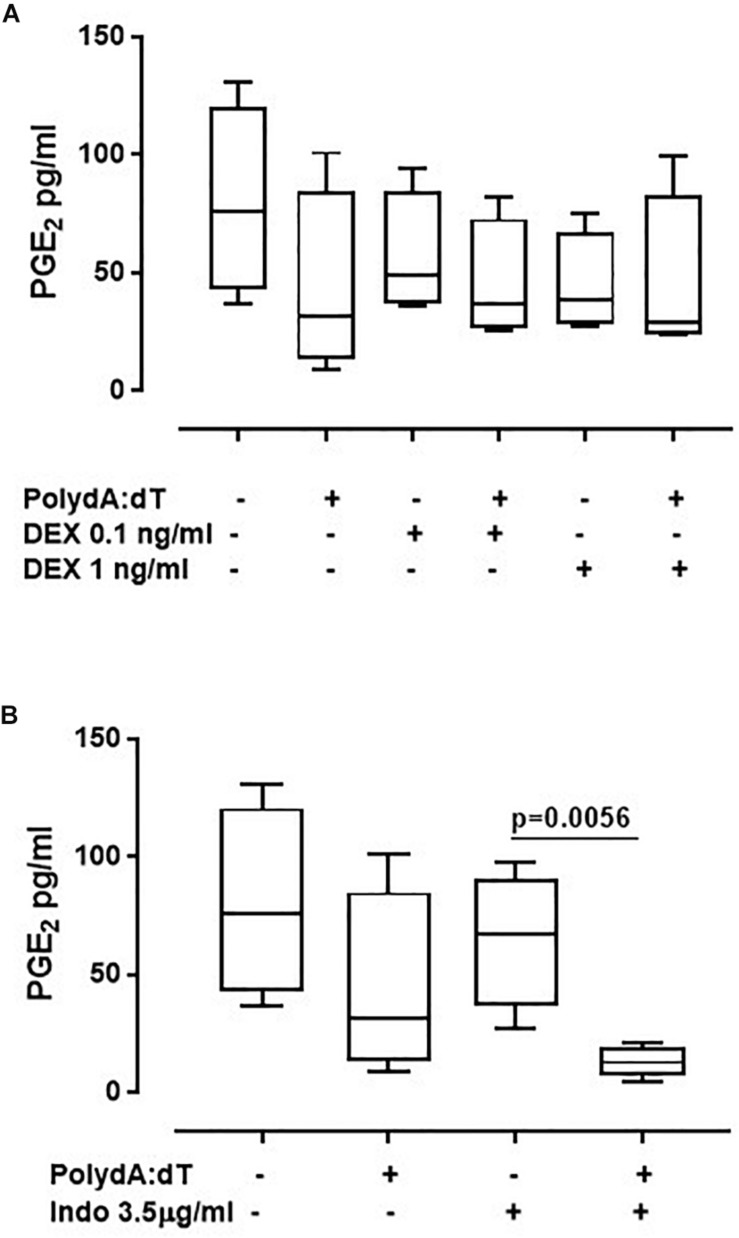
Corticosteroids treatment did not affect the release of PGE_2_ from exacerbated COPD-derived PBMCs. Exacerbated COPD-derived PBMCs were stimulated with Dexamethasone (DEX, 0.1 ng/ml and 1 ng/ml) **(A)** and Indomethacin (Indo, 3.5 μg/ml) **(B)** Poly dA:dT (1 μg/ml), and PGE_2_ levels were evaluated. Data are represented as median ± interquartile range (*n* = 4). Statistically significant differences were determined by ONE-way ANOVA followed by Dunn’s post–test.

To further understand whether the corticosteroids-dependent downstream pathway was altered after AIM2 activation, we analyzed the levels of Prostaglandin E_2_ (PGE_2_). The stimulation of AIM2 for 5 h by means of Poly dA:dT did not increase the levels of PGE_2_ in exacerbated COPD-derived PBMCs (Figure 4A). Instead, it induced a downward trend of PGE_2_ levels, although not in a significant manner (Non-treated: 80 ± 13.2 pg/ml vs. Poly dA:dT: 43.3 ± 13.12 pg/ml) (Figure 4A). Similar data were observed for stable COPD-derived PBMCs (CTR: 113 ± 10.1 pg/ml vs. Poly dA:dT: 39.4 ± 1 pg/ml). In support, the administration of both DEX (Figure 4A) and Indo (Figure 4B) did not alter the release of PGE_2_.

Altogether, these data imply that AIM2 inflammasome-dependent IL-1α release is not correlated to the release of eicosanoids from PBMCs obtained from both exacerbated and stable COPD patients; rather, another novel mechanism, AIM2-dependent, may be responsible for the inflammatory pattern that leads to the exacerbation phase of COPD.

### Corticosteroids Did Not Alter AIM2-Dependent TGF-β Release From Exacerbated COPD-Derived PBMCs

**FIGURE 5:**
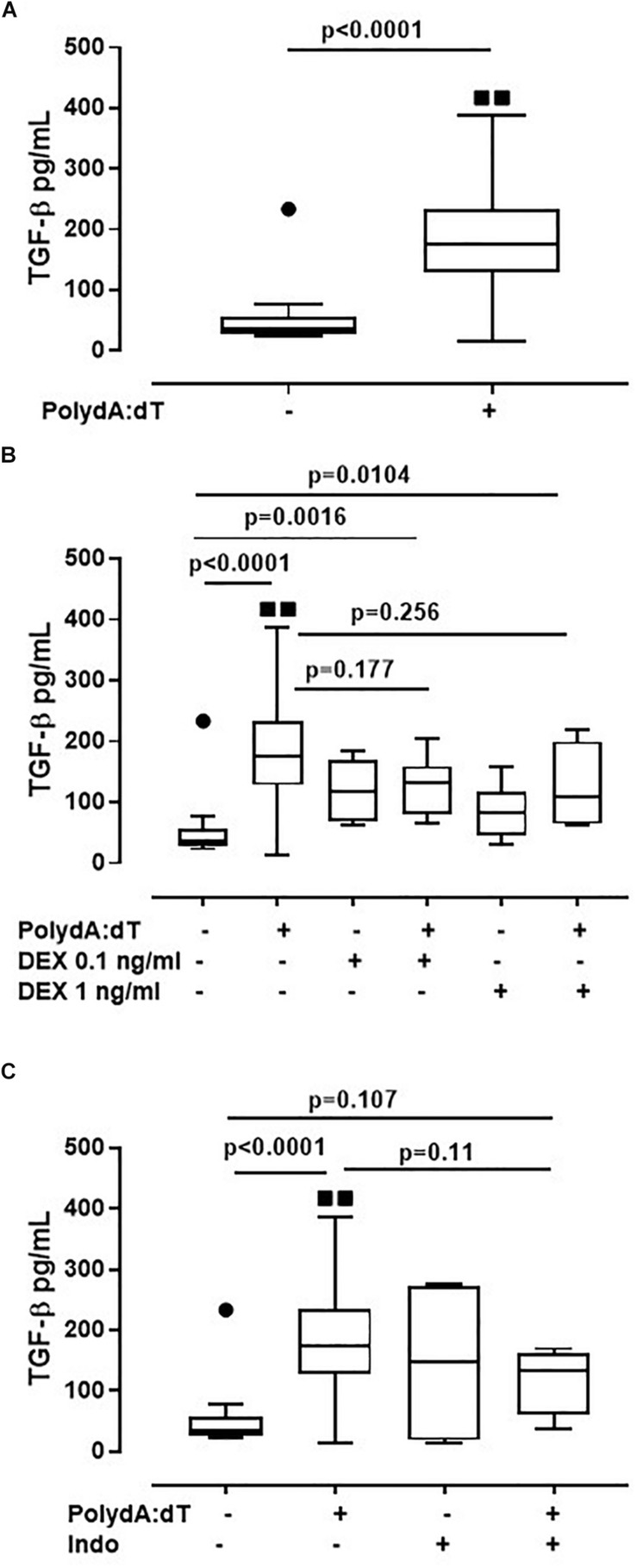
Corticosteroids did not alter AIM2-dependent TGF-β release from exacerbated COPD-derived PBMCs. **(A)** AIM2 triggered with Poly dA:dT (1 μg/ml), significantly increased the release of TGF-β after 24 h of treatment from exacerbated COPD-derived PBMCs. **(B)** Dexamethasone (DEX, 0.1 ng/ml and 1 ng/ml) treatment, in the presence of Poly dA:dT, did not alter TGF-β release. Similarly, no differences were observed when exacerbated COPD-derived PBMCs were treated with Indomethacin (Indo, 3.5 μg/ml) in the presence or absence of Poly dA:dT **(C)**. Data are represented as median ± interquartile range (*n* = 14). Statistically significant differences were determined by ONE-way ANOVA followed by Dunn’s post–test.

Because the activation of the AIM2/caspase-1/caspase-4/IL-1α axis is responsible for the release of TGF-β from exacerbated COPD-derived PBMCs, in this study, we evaluated the effect of corticosteroids on AIM2 inflammasome-dependent TGF-β release. The activation of AIM2 significantly increased the release of TGF-β after 24 h of treatment from exacerbated COPD-derived PBMCs (Figure 5A). Again, the administration of DEX (0.1 ng/ml and 1 ng/ml) on Poly dA:dT-treated PBMCs did not reduce the release of TGF-β (Figure 5B). Similarly, treatment with Indo did not alter the release of TGF-β (Figure 5C). It is worth noting that stable COPD-derived PBMCs did not show any increase in TGF-β after AIM2 activation, a condition that was not altered after the addition of DEX or Indo (Supplementary Figure S1).

These data highlight that the AIM2/IL-1α/TGF-β axis is not altered by the administration of Dexamethasone, implying that this biological pathway is not monitored from the actual therapeutic options used for COPD patients.

## Discussion

In our recent study, we found that the axis AIM2/caspase-1/caspase-4/IL-1α was responsible for TGF-β release (Colarusso et al., 2019), involved in COPD exacerbation (Mak et al., 2009; Colarusso et al., 2019). Starting from the above published data, we found that PBMCs obtained from exacerbated, but not stable, COPD patients were more responsive to AIM2 activation regardless of corticosteroid treatment, implying that AIM2 plays a role during the exacerbation phase of COPD. COPD patients are routinely treated with LABAs, bronchodilators and corticosteroids, but the exacerbation status still occurs, implying that, despite the administration of potent immunosuppressive drugs, such as corticosteroids, other inflammatory pathways are activated or are out of control during the exacerbation phase, which is clinically deleterious for the patient. Because corticosteroids are well-known to induce IL-1 Receptor Antagonist (IL-1RA) transcription (Levine et al., 1996), which binds to both IL-1α and IL-1β, we believed that the administration of corticosteroids was able to reduce IL-1α release, which we demonstrated to be responsible for TGF-β release during the exacerbation phase of COPD (Colarusso et al., 2019). Surprisingly, the administration of Dexamethasone onto exacerbated, but not stable, COPD-derived PBMCs was not able to abolish AIM2-dependent IL-1α release, implying that corticosteroids treatment does not influence AIM2-dependent inflammatory signaling.

This mechanism could explain why during treatment with corticosteroids, COPD exacerbation still progresses, leading to worsened respiratory functionality. In particular, the AIM2/IL-1α axis may be responsible for the inflammatory pattern which enhances the exacerbation process in an eicosanoid-independent manner, which explains why corticosteroids treatment is not able to control the exacerbation induction. In support, the expression of AIM2 in COPD-derived CD14^+^ cells was higher in 75% of pair-matched samples obtained from the same patients who underwent from the exacerbation to the stable status. These data may suggest that corticosteroids treatment during hospitalization may increase AIM2 expression, without increasing its activity in that the stimulation of this receptor in a stable condition did not increase IL-1α release in the same manner as observed for exacerbated patients, implying that AIM2 is not active in stable COPD patients. These data are in line with those from other researchers in that inflammasome components, such as caspase-1 and ASC, regardless of the expression of the upstream receptors (Faner et al., 2016), are in their inactive form, suggesting that AIM2 activity could be negatively regulated during the stable status of COPD. However, more studies are needed to understand how and why AIM2 may be negatively regulated during stable conditions.

In support of AIM2 inactivity during stable disease, no differences in terms of TGF-β release were observed in stable COPD-derived PBMCs at basal conditions and after Poly dA:dT addition. In sharp contrast, the stimulation of AIM2 significantly increased the levels of TGF-β from exacerbated COPD-derived PBMCs. An important issue is that the administration of corticosteroids still did not reduce the levels of TGF-β after AIM2 stimulation, further supporting our hypothesis that the activation of AIM2 occurs independently of the eicosanoid’s pathway. In this regard, the activation of AIM2 did not increase PGE_2_ levels. It is known that TGF-β levels are high in the plasma and in different compartments of the lower airway of COPD patients compared to healthy subjects, and that it is an important mediator in the pathogenesis of COPD (Mak et al., 2009; Di Stefano et al., 2018). Although only local minor fibrotic lesions appear in the lung of COPD patients, TGF-β regulates cell proliferation, differentiation, extracellular matrix synthesis and apoptosis, which are all important processes in COPD pathogenesis (Di Stefano et al., 2018). These results propose a novel mechanism associated with the release of TGF-β during COPD exacerbation that is not counteracted by corticosteroid treatment, and that contributes to disease worsening and progression.

Several studies have demonstrated that IL-1α together with TGF-β can play a crucial role in lung inflammation and fibrosis (Sorrentino et al., 2015; Terlizzi et al., 2016, 2018). In this study we found that the activation of AIM2, independently of glucocorticoid treatment, still induces PBMCs to participate in fibrotic processes of the airway wall with consequential luminal narrowing and airflow limitation.

These unexpected data could be explained by oxidative stress which is involved in many molecular mechanisms and drives many features of COPD (Colarusso et al., 2017; De Falco et al., 2017a). It is known that oxidative stress reduces the efficacy of corticosteroids by decreasing levels of histone deacetylase-2 (HDAC2) (Roche et al., 2011). To note, all of our patients were smokers or former smokers (Table 1). Cigarette smoke, through peroxynitrite formation, generates oxidative stress, impairing HDAC2 activity, and consequently amplifying the inflammatory response to NF-κB activation, resulting in a reduced anti-inflammatory effect of corticosteroids (Barnes and Adcock, 2009). Previously, we found that COPD-derived PBMCs had higher basal levels of 8-hydroxy-2′-deoxyguanosine (8-OH-dG), a marker of oxidative stress to DNA, that further increased when AIM2 was stimulated (Colarusso et al., 2019), compared to healthy smokers and non-smokers subjects-derived PBMCs that, accordingly, showed higher levels of 8-Oxoguanine glycosylase (OGG1), a repairing DNA enzyme (De Falco et al., 2017a). Therefore, we could speculate that AIM2-dependent oxidative stress may be involved in the inflammatory pattern which enhances the exacerbation process, even under corticosteroid treatment, in that exacerbated COPD-derived PBMCs were not responsive to Dexamethasone, allowing the release of IL-1α and thus amplifying the inflammatory pattern in exacerbated COPD patients. Therefore, these results, in line with our previous data, highlight a new pro-inflammatory mechanism involved in COPD exacerbation. It is worth noting that the administration of Dexamethasone onto exacerbated, but not stable, COPD-derived PBMCs was not able to abolish AIM2-dependent IL-1α release, implying that corticosteroid treatment does not influence AIM2-dependent inflammatory signaling, which could explain why during the treatment with corticosteroids, COPD exacerbation still progresses, leading to worsened respiratory functionality.

## Conclusion

In conclusion, although the link that correlates the patterns of lung inflammation to the clinical outcomes, response to therapy, and prognosis of COPD patients is still very elusive, our study proposes a novel AIM2 inflammasome-dependent mechanism that could be involved in the failure of corticosteroid therapy, taking an important step forward and opening new horizons to alternative treatment strategies for COPD patients.

## Data Availability Statement

All datasets generated for this study are included in the manuscript/Supplementary Files.

## Ethics Statement

The studies involving human participants were reviewed and approved by the Ethical Committee of the “Monaldi-Azienda Ospedaliera (AORN)-Ospedale dei Colli” (protocol n. 604/2017). The patients/participants provided their written informed consent to participate in this study.

## Author Contributions

MT, CC, and AR performed the experiments. AM, AS, and PS performed the diagnostic analyses. AP edited the manuscript. RS designed the experiments, and analyzed and interpreted the data. MT wrote the manuscript.

## Conflict of Interest

The authors declare that the research was conducted in the absence of any commercial or financial relationships that could be construed as a potential conflict of interest.

## Supplementary Material

FIGURE S1 TGF-β release from stable COPD-derived PBMCs. No differences were observed in stable patients after Dexamethasone (DEX, 0.1 ng/ml and 1 ng/ml) and Indomethacin (Indo, 3.5 μg/ml) treatment, in the presence or absence of Poly dA:dT (dA, 1 μg/ml). Data are represented as median ± interquartile range (*n* = 5).

## References

[B1] Barnes P. J. (2013). Corticosteroid resistance in patients with asthma and chronic obstructive pulmonary disease. *J. Allergy Clin. Immunol.* 131 636–645. 10.1016/j.jaci.2012.12.1564 10.1016/j.jaci.2012.12.1564 23360759

[B2] Barnes P. J. (2016). Inflammatory mechanisms in patients with chronic obstructive pulmonary disease. *J. Allergy Clin. Immunol.* 138 16–27. 10.1016/j.jaci.2016.05.011 27373322

[B3] Barnes P. J. Adcock I. M. (2009). Glucocorticoid resistance in inflammatory diseases. *Lancet* 373 1905–1917. 10.1016/S0140-6736(09)60326-3 19482216

[B4] Bhat T. A. Panzica L. Kalathil S. G. Thanavala Y. (2015). Immune dysfunction in patients with chronic obstructive pulmonary disease. *Ann. Am. Thorac. Soc.* 12(Suppl. 2), S169–S175. 10.1513/AnnalsATS.201503-126AW 26595735 PMC4722840

[B5] Borchers M. T. Wesselkamper S. C. Harris N. L. Deshmukh H. Beckman E. Vitucci M. (2007). CD8+ T cells contribute to macrophage accumulation and airspace enlargement following repeated irritant exposure. *Exp. Mol. Pathol.* 83 301–310. 10.1016/j.yexmp.2007.08.020 17950725 PMC2140237

[B6] Colarusso C. Terlizzi M. Molino A. Imitazione P. Somma P. Rega R. (2019). AIM2 inflammasome activation leads to IL-1α and TGF-β release from exacerbated chronic obstructive pulmonary disease-derived peripheral blood mononuclear cells. *Front. Pharmacol.* 10:257 10.3389/fphar.2019.00257 PMC6428726 30930781

[B7] Colarusso C. Terlizzi M. Molino A. Pinto A. Sorrentino R. (2017). Role of the inflammasome in chronic obstructive pulmonary disease (COPD). *Oncotarget* 8 81813–81824. 10.18632/oncotarget.17850 29137224 PMC5669850

[B8] De Falco G. Colarusso C. Terlizzi M. Popolo A. Pecoraro M. Commodo M. (2017a). Chronic obstructive pulmonary disease-derived circulating cells release IL-18 and IL-33 under ultrafine particulate matter exposure in a caspase-1/8-independent manner. *Front. Immunol.* 8:1415. 10.3389/fimmu.2017.01415 29123531 PMC5662642

[B9] De Falco G. Terlizzi M. Sirignano M. Commodo M. D’Anna A. Aquino R. P. (2017b). Human peripheral blood mononuclear cells (PBMCs) from smokers release higher levels of IL-1-like cytokines after exposure to combustion-generated ultrafine particles. *Sci. Rep.* 7:43016. 10.1038/srep43016 28223692 PMC5320442

[B10] De Nardo D. De Nardo C. M. Latz E. (2014). New insights into mechanisms controlling the NLRP3 inflammasome and its role in lung disease. *Am. J. Pathol.* 184 42–54. 10.1016/j.ajpath.2013.09.007 24183846 PMC3873477

[B11] Di Stefano A. Sangiorgi C. Gnemmi I. Casolari P. Brun P. Ricciardolo F. L. M. (2018). TGF-β signaling pathways in different compartments of the lower airways of patients with stable COPD. *Chest* 153 851–862. 10.1016/j.chest.2017.12.017 29289685 PMC5883327

[B12] Faner R. Sobradillo P. Noguera A. Gomez C. Cruz T. López-Giraldo A. (2016). The inflammasome pathway in stable COPD and acute exacerbations. *ERJ Open Res.* 2:00002-2016. 10.1183/23120541.00002-2016 27730204 PMC5034597

[B13] Fujimoto K. Yasuo M. Urushibata K. Hanaoka M. Koizumi T. Kubo K. (2005). Airway inflammation during stable and acutely exacerbated chronic obstructive pulmonary disease. *Eur. Respir. J.* 25 640–646. 10.1183/09031936.05.00047504 15802337

[B14] Guo Q. Wu Y. Hou Y. Liu Y. Liu T. Zhang H. (2018). Cytokine secretion and pyroptosis of thyroid follicular cells mediated by enhanced NLRP3, NLRP1, NLRC4, and AIM2 inflammasomes are associated with autoimmune thyroiditis. *Front. Immunol.* 9:1197. 10.3389/fimmu.2018.01197 29915579 PMC5994487

[B15] Huang G. Xu X. C. Zhou J. S. Li Z. Y. Chen H. P. Wang Y. (2017). Neutrophilic inflammation in the immune responses of chronic obstructive pulmonary disease: lessons from animal models. *J. Immunol. Res.* 2017:7915975. 10.1155/2017/7915975 28536707 PMC5426078

[B16] Levine S. J. Benfield T. Shelhamer J. H. (1996). Corticosteroids induce intracellular interleukin-1 receptor antagonist type I expression by a human airway epithelial cell line. *Am. J. Respir. Cell Mol. Biol.* 15 245–251. 10.1165/ajrcmb.15.2.8703481 8703481

[B17] Mak J. C. Chan-Yeung M. M. Ho S. P. Chan K. S. Choo K. Yee K. S. (2009). Elevated plasma TGF-beta1 levels in patients with chronic obstructive pulmonary disease. *Respir. Med.* 103 1083–1089. 10.1016/j.rmed.2009.01.005 19186046

[B18] Miravitlles M. Vogelmeier C. Roche N. Halpin D. Cardoso J. Chuchalin A. G. (2016). A review of national guidelines for management of COPD in Europe. *Eur. Respir. J.* 47 625–637. 10.1183/13993003.01170-2015 26797035 PMC4733567

[B19] Roche N. Marthan R. Berger P. Chambellan A. Chanez P. Aguilaniu B. (2011). Beyond corticosteroids: future prospects in the management of inflammation in COPD. *Eur. Respir. Rev.* 20 175–182. 10.1183/09059180.00004211 21881145 PMC9584116

[B20] Sorrentino R. Terlizzi M. Di Crescenzo V. G. Popolo A. Pecoraro M. Perillo G. (2015). Human lung cancer-derived immunosuppressive plasmacytoid dendritic cells release IL-1α in an AIM2 inflammasome-dependent manner. *Am. J. Pathol.* 185 3115–3124. 10.1016/j.ajpath.2015.07.009 26506473

[B21] Sugimoto M. A. Sousa L. P. Pinho V. Perretti M. Teixeira M. M. (2016). Resolution of inflammation: what controls its onset? *Front. Immunol.* 7:160. 10.3389/fimmu.2016.00160 27199985 PMC4845539

[B22] Tashkin D. P. Strange C. (2018). Inhaled corticosteroids for chronic obstructive pulmonary disease: what is their role in therapy? *Int. J. Chron. Obstruct. Pulmon. Dis.* 13 2587–2601. 10.2147/COPD.S172240 30214177 PMC6118265

[B23] Terlizzi M. Casolaro V. Pinto A. Sorrentino R. (2014). Inflammasome: cancer’s friend or foe? *Pharmacol. Ther.* 143 24–33. 10.1016/j.pharmthera.2014.02.002 24518102

[B24] Terlizzi M. Colarusso C. Popolo A. Pinto A. Sorrentino R. (2016). IL-1α and IL-1β-producing macrophages populate lung tumor lesions in mice. *Oncotarget* 7 58181–58192. 10.18632/oncotarget.11276 27528423 PMC5295423

[B25] Terlizzi M. Molino A. Colarusso C. Donovan C. Imitazione P. Somma P. (2018). Activation of the absent in melanoma 2 inflammasome in peripheral blood mononuclear cells from idiopathic pulmonary fibrosis patients leads to the release of pro-fibrotic mediators. *Front. Immunol.* 9:670. 10.3389/fimmu.2018.00670 29675024 PMC5895962

[B26] Wang H. Lv C. Wang S. Ying H. Weng Y. Yu W. (2018). NLRP3 Inflammasome involves in the acute exacerbation of patients with chronic obstructive pulmonary disease. *Inflammation* 41 1321–1333. 10.1007/s10753-018-0780-0 29656319

[B27] Wang Y. Xu J. Meng Y. Adcock I. M. Yao X. (2018). Role of inflammatory cells in airway remodeling in COPD. *Int. J. Chron. Obstruct. Pulmon. Dis.* 13 3341–3348. 10.2147/COPD.S176122 30349237 PMC6190811

