# Supplementary figures and images for: AIM2/IL-1α/TGF-β Axis in PBMCs From Exacerbated Chronic Obstructive Pulmonary Disease (COPD) Patients Is Not Related to COX-2-Dependent Inflammatory Pathway
Source: Front Physiol. 2019 Oct 1;10:1235. doi: 10.3389/fphys.2019.01235 (PMC6780005; doi:10.3389/fphys.2019.01235)

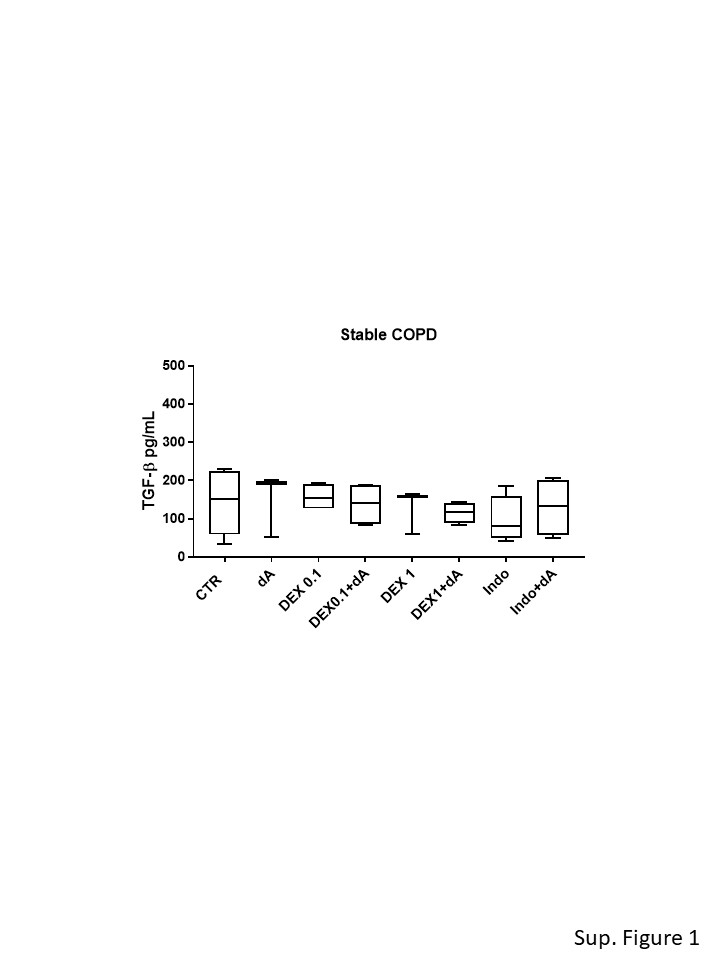

Supplement: FIGURE S1 — TGF-β release from stable COPD-derived PBMCs. No differences were observed in stable patients after Dexamethasone (DEX, 0.1 ng/ml and 1 ng/ml) and Indomethacin (Indo, 3.5 μg/ml) treatment, in the presence or absence of Poly dA:dT (dA, 1 μg/ml). Data are represented as median ± interquartile range (n = 5). [file Image_1.jpg]
